# Subjective memory impairment after electroconvulsive therapy – potentially modifiable?

**DOI:** 10.1192/bjo.2020.20

**Published:** 2020-04-06

**Authors:** Kate Eggleston, Richard Porter

**Affiliations:** Canterbury District Health Board New Zealand; and Department of Psychological Medicine, University of Otago, Christchurch, New Zealand; Canterbury District Health Board, New Zealand; and Department of Psychological Medicine, University of Otago, Christchurch, New Zealand

**Keywords:** Electroconvulsive therapy, memory, cognition

## Abstract

Memory impairment is an important side-effect of electroconvulsive therapy (ECT). However, predicting which patients are at increased risk of developing this is difficult. The study by Sigström et al compares patients’ experience of memory difficulties before and after ECT and suggests that patients with negative expectations of ECT's memory effects are more likely to have subjective memory worsening post-ECT. This intriguing finding suggests that clinicians may be able to modify the risk of patients developing subjective memory difficulties post-ECT by providing appropriate information and addressing concerns prior to treatment, during the informed consent process.

Electroconvulsive therapy (ECT) is a highly effective treatment, however, it may lead to cognitive side-effects, with memory impairment one of the most common and distressing effects. Although it is well established that memory impairment is more significant immediately following ECT, the longer-term memory effects are less clear. Information regarding predictors of increased risk of memory impairment is of importance to both patients and clinicians, and may assist with treatment decisions. Additionally, if these risk factors are modifiable, this could provide opportunities to reduce the risk of memory impairment after ECT.

Sigström et al^1^ present findings on longer-term subjective memory impairment after ECT, using national registry data prior to and following treatment. The study forms part of a larger project investigating the predictors of response to and side-effects of ECT. Interestingly, in most (83.7%) participants, subjective memory (measured using the Comprehensive Psychopathological Rating Scale) either remained unchanged or improved. In contrast to this, over half of participants (54.5%) reported a negative effect when they were asked to rate the effect of ECT on their memory using a simple seven-point Likert scale (the Global Self Evaluation (GSE) of Memory). Subjective memory worsening was associated with more negative expectations of the likely memory effects of ECT before treatment, along with younger age and shorter duration of follow-up. However, when subjective assessment of current memory difficulties was compared between baseline and post-ECT, only 16.2% were found to have worsened.

These findings are of practical importance for a number of reasons. First, the availability of data regarding subjective memory impairment both prior to and following ECT is in contrast to several other studies that only examine this post-ECT. This provides much more meaningful information. Second, unlike many ECT studies, patients taking concomitant medications were included, increasing the applicability of this data to clinical settings. Third, in previous ECT memory research, with notable exceptions,^2^ follow-up has often been short term, meaning that longer-term memory effects of treatment are less clear. In this study, the median post-ECT assessment of memory was 73 days. Although this still does not provide very long-term information, the finding that shorter duration of follow-up was significantly associated with poorer subjective memory suggests that memory may improve further over time.

## Relationship of subjective and objective measures of cognitive impairment post-ECT

Autobiographical memory impairment is an important side-effect of ECT, and is perceived as persistent by some patients.^3^ The majority of contemporary ECT studies have used the Colombia University Autobiographical Memory Interview (CUAMI) or its shorter form (CUAMI-SF) as an objective measure of this. The relationship between objective and subjective assessment of autobiographical memory impairment has been studied. Brakemeier et al^4^ demonstrated that longer and more detailed scales measuring subjective memory impairment (Cognitive Failures Questionnaire and Subjective Memory Complaints Questionnaire) did not correlate well with the CUAMI. However, there was a high level of correlation between the CUAMI and the GSE of Memory (one of the questionnaires used by Sigström et al).^4^ The GSE of Memory consists of two simple questions where patients are asked to rate their expectations about the likely effect of ECT on their memory prior to treatment, and post-ECT to evaluate the overall effect that it has had on memory. This is similar to the common clinical practice of assessing patients’ memory by asking them about it directly. The correlation between this simple scale and a more detailed assessment of autobiographical memory indicates that patient experience of cognitive impairment may be more important, and closer to objective measures of memory, than is sometimes suggested. It also suggests that the data presented by Sigström et al, particularly using the GSE of Memory, may relate both to subjective and objective memory difficulties.

## Limitations of the data

There are several caveats to the findings presented in this study. Missing data has been a difficult issue in ECT research, with many studies reporting around a 50% drop-out rate.^2^

Similarly, in this study approximately 48% of participants were not able to be included in the final analysis, the majority because of a lack of post-ECT data regarding memory measures. One important possibility is that patients with more significant cognitive or memory impairment may be less likely to undertake further assessment, leading to an underestimate of the effect of ECT on memory. Similarly, patients with more severe depressive symptoms may be less likely to engage in ongoing testing, and may have more ongoing cognitive symptoms, including memory impairment, than those who experience an improvement in these symptoms with ECT.

Clinical features may also contribute to the presence of subjective memory impairment following ECT. For instance, patients with more severe depressive symptoms may be at higher risk of having or developing cognitive impairment. They may also be more likely to undergo ECT. This makes delineating the effects of ECT on cognition difficult. Similarly, the negativity typically associated with more severe depression may have led participants to report more negative expectations of treatment. It would have been helpful to measure depression severity and in future research this could be controlled for. Data were not presented on the correlations between the GSE of Memory and the GSE of Mood, which would have assisted in examining these potential associations.

In addition to clinical factors, there is some evidence that rates of memory difficulties following ECT may depend on the assessor and location of testing. Lower rates of subjective memory impairment have been reported in studies where medical assessors in clinical settings were used.^5^ In the current study, pre-ECT subjective memory was assessed in person, while post-ECT effects were measured over the phone. This may have had some impact on how participants responded to questions regarding subjective memory, and as such the responses given post-ECT may have been more negative, and possibly more realistic, than those given in person prior to treatment.

## Clinical implications

Despite the limitations and the subjective nature of the assessments, this data provides important information regarding which patients are more likely to experience memory impairment following ECT. In particular, subjective memory worsening was associated with more negative expectations of the likely memory effects of ECT before treatment, something that could potentially be modified. Interestingly, patients’ expectations of mood effects of ECT were not associated with negative effects on memory. This suggests that the finding of an association between negative expectations of ECT and subjective memory impairment does not relate to a general negative attitude towards ECT. A number of factors may have an impact on patients’ attitudes and expectations about treatment with ECT, including the manner in which ECT is discussed.

There is some suggestion that attempting to downplay the cognitive side-effects of ECT may be unhelpful, and can invalidate patients’ experience of subjective cognitive impairment.^6^ On the other hand, a discussion where cognitive side-effects are highlighted as likely to occur may increase the chances that patients expect, and possibly develop memory impairment. The consent process presents an opportunity for clinicians to modify patients' expectations of ECT, its effects and potential side-effects. A balanced discussion, that acknowledges but does not minimise side-effects, may benefit subjective memory outcomes.

Overall, this prospective study provides some reassurance regarding the longer-term memory effects of ECT, with only a small proportion (16.2%) of patients found to have worsening in subjective memory compared with prior to ECT. It adds to the growing literature regarding the importance of subjective memory impairment after ECT, and we suggest that measurement of subjective memory should be a routine part of treatment and follow-up. The intriguing finding that those patients who anticipated negative effects of ECT on memory were more likely to have higher rates of subjective memory impairment at follow-up indicates that further research into identifying these patients is required. It also implies that subjective memory impairment after ECT may be modifiable, possibly by addressing negative expectations prior to treatment, or by adjusting the way in which ECT is discussed with patients. In future, it would be of interest to trial a specific education or a consistent informed consent process, to determine whether this has a positive impact on subjective memory impairment and its associated distress. In the meantime, we suggest that clinicians take a balanced approach to discussing the possible memory effects of ECT with patients. By exploring their patients’ expectations and understanding of ECT, and addressing negative expectations prior to treatment, there may be an opportunity for clinicians to modify their subjective memory outcomes.

## Supplementary material

For supplementary material accompanying this paper visit https://doi.org/10.1192/bjo.2020.20. click here to view supplementary material
